# [Retracted] Hsp72 controls bortezomib-induced HepG2 cell death via interaction with pro-apoptotic factors 

**DOI:** 10.3892/or.2026.9133

**Published:** 2026-05-11

**Authors:** Giuseppe Calvaruso, Michela Giuliano, Patrizia Portanova, Ornella Pellerito, Renza Vento, Giovanni Tesoriere

Oncol Rep 18: 447–450, 2007; DOI: 10.3892/or.18.2.447

Subsequently to the publication of the above article, a concerned reader drew the Editor's attention to the fact that specific protein bands featured within the western blots in Figs. 1A, 2B and 4 were strikingly similar. Having assessed these data independently in the Editorial Office, some of the concerns raised by the reader were judged to have been well founded; in particular, the striking similarity of the protein bands shown for the IP AIG + and IP Apaf-1 + experiments in Fig. 2B, and of a pair of β-actin bands within the blot for Fig. 1A, such that these striking similarities in the appearance of the abovementioned protein bands, accompanied by the surrounding gel backgrounds, could not easily be attributed to coincidence. Therefore, in view of the probable anomalies that have been identified in this paper, the Editor of *Oncology Reports* has decided that it should be retracted from the Journal on account of a lack of confidence in the presented data. The authors were asked for an explanation to account for these concerns, but the Editorial Office did not receive a reply. The Editor apologizes to the readership for any inconvenience caused.

